# Pangenome and multi-tissue gene atlas provide new insights into the domestication and highland adaptation of yaks

**DOI:** 10.1186/s40104-024-01027-2

**Published:** 2024-05-06

**Authors:** Daoliang Lan, Wei Fu, Wenhui Ji, Tserang-Donko Mipam, Xianrong Xiong, Shi Ying, Yan Xiong, Peng Sheng, Jiangping Ni, Lijun Bai, Tongling Shan, Xiangdong Kong, Jian Li

**Affiliations:** 1 Ministry of Education of Key Laboratory of Qinghai-Tibetan Plateau Animal Genetic Resource and Utilization, Southwest Minzu University, Chengdu, China; 2 College of Animal & Veterinary Sciences, Southwest Minzu University, Chengdu, China; 3 Institute of Qinghai-Tibetan Plateau, Southwest Minzu University, Chengdu, China; 4 Jiguang Gene Biotechnology Co., Ltd., Nanjing, China; 5 Chengdu Genepre Technology Co., Ltd., Chengdu, China; 6 Shanghai Veterinary Research Institute, Chinese Academy of Agricultural Sciences, Shanghai, China

**Keywords:** High- and low-altitude, Novel genes, Pangenome, PAV-GWAS, Yak

## Abstract

**Background:**

The genetic diversity of yak, a key domestic animal on the Qinghai-Tibetan Plateau (QTP), is a vital resource for domestication and breeding efforts. This study presents the first yak pangenome obtained through the de novo assembly of 16 yak genomes.

**Results:**

We discovered 290 Mb of nonreference sequences and 504 new genes. Our pangenome-wide presence and absence variation (PAV) analysis revealed 5,120 PAV-related genes, highlighting a wide range of variety-specific genes and genes with varying frequencies across yak populations. Principal component analysis (PCA) based on binary gene PAV data classified yaks into three new groups: wild, domestic, and Jinchuan. Moreover, we proposed a ‘two-haplotype genomic hybridization model’ for understanding the hybridization patterns among breeds by integrating gene frequency, heterozygosity, and gene PAV data. A gene PAV-GWAS identified a novel gene (BosGru3G009179) that may be associated with the multirib trait in Jinchuan yaks. Furthermore, an integrated transcriptome and pangenome analysis highlighted the significant differences in the expression of core genes and the mutational burden of differentially expressed genes between yaks from high and low altitudes. Transcriptome analysis across multiple species revealed that yaks have the most unique differentially expressed mRNAs and lncRNAs (between high- and low-altitude regions), especially in the heart and lungs, when comparing high- and low-altitude adaptations.

**Conclusions:**

The yak pangenome offers a comprehensive resource and new insights for functional genomic studies, supporting future biological research and breeding strategies.

**Supplementary Information:**

The online version contains supplementary material available at 10.1186/s40104-024-01027-2.

## Background

Yak (*Bos grunniens*), known as the ‘plateau ship’, is a unique species of livestock distributed mainly in the Qinghai-Tibetan Plateau (QTP) and nearby alpine or subalpine regions [1]. Yaks are well suited to alpine grassland environments and survive and reproduce under challenging conditions. Locally, yaks are highly important because they provide milk, meat, wool, labor, and fuel to pastoralists in the region [2]. There are approximately 17.6 million recorded yaks worldwide [3]. While domestic yaks have adapted to different morphological structures, production capabilities, and geographical environments, they share a common ancestry with wild yaks, despite diverging approximately 4.9 million years ago. It has been demonstrated that interspecific hybridization between *Bos taurus* and *Bos grunniens* can give rise to fertile offspring [4], and there is evidence of gene flow between these two species [5]. These hybridization events contribute to genetic diversity within the yak population, which is a valuable resource for domestication, modern breeding, and future genetic improvements to meet the increasing food demand on the QTP.

Due to advancements in high-throughput sequencing technologies, multiple editions of the yak reference genome have been released within the last decade [1, 6–8]. Furthermore, numerous samples of domestic and wild yaks have been resequenced [3, 9–12]. These investigations have played a vital role in revealing genomic alterations and their significance for the evolutionary trajectory, domestication processes, and phenotypic characteristics of yaks. Additionally, an extensive repertoire of mitochondrial genomes from diverse yaks has been documented [13–17], offering a valuable resource for studies based on mitochondrial genomes. Several transcriptome studies have shed light on the potential involvement of candidate genes, lncRNAs, and circRNAs in shaping the environmental adaptability and phenotypic traits of yaks through the modulation of gene expression [18–21].

However, resequencing or transcriptome studies currently rely on a single yak reference genome. In these studies, the identification of genomic variation was achieved through the alignment of short reads to the reference genome, potentially leading to the loss of information from highly polymorphic regions [22, 23]. Moreover, functionally significant genes may be absent in the reference genome following the assembly of six yak genomes using second-generation whole-genome sequencing data [6]. Recently, 29 yak individuals were resequenced using third-generation sequencing (TGS); although 372,220 structural variations were identified [7], no information regarding novel or lost genes within the population was obtained. These studies indicate that one or a few yak genomes cannot include the entire spectrum of important genomic content and cannot fully represent the genetic diversity of the species.

The pangenome represents the gene set of a species rather than an individual, allowing the discovery of genetic diversity and variation that may be missed by using a single reference genome [22–24]. It is also useful for detecting presence/absence variations (PAVs) that cannot be identified based on single nucleotide polymorphisms (SNPs) and for exploring the distribution of these variants and potential new genes at the population level [25, 26].

In this study, we performed de novo assembly of 16 yak genomes using TGS data and constructed a yak pangenome with these assembled genomes and resequencing data from 350 yaks. The pangenome captured 290 Mb of nonreference sequences and 504 novel genes were identified. By using the pangenome as a reference, we identified 5,120 genes with gene PAVs in more than 1% of the population. We also identified breed-specific genes and gene PAVs under selection between different populations. Furthermore, we proposed a novel analysis method using the “two-haplotype genomic hybridization model” to determine the hybridization track between breeds based on the frequency and heterozygosity results of certain genes in different populations and the fusion of PAV information. This method contributes to the accurate discovery and understanding of interspecific hybridization in yaks and the evaluation of germplasm resources. Analysis of the pangenome-wide PAV gene revealed new yak population structures and identified 107 genes specific to Changtai and Maiwa yaks, as well as high- and low-frequency genes in wild or domesticated yaks. Gene PAV-GWAS identified a novel gene, BosGru3G009179 (phosphatidylinositol N-acetylglucosaminyltransferase subunit H), which may be associated with the multirib trait in Jinchuan yaks. We performed integrated transcriptome analysis using the pangenome, combining PAVs and SNPs. This analysis revealed high expression of core genes and differentially expressed genes between high- and low-altitude yaks. Additionally, transcriptome analysis of multiple species revealed that yaks possess the most differentially expressed mRNAs and long noncoding RNAs specifically expressed in the heart and lungs. Overall, this study provides valuable resources and novel insights for genomic studies on yaks, particularly for understanding genomic variation, population structure, plateau adaptation, and traits. The establishment of a yak pangenome will be beneficial for utilizing various alleles within gene pools for further breeding.

## Methods

### DNA sequencing data retrieval and genome assembly

Sixteen sets of high-quality yak data were selected and retrieved from the recently published data of 6 wild and 23 domestic yaks (NCBI: PRJNA540974) [7]. The sequencing of these 29 yak samples generated Oxford Nanopore long reads, with sequencing depths ranging from 8.4 to 15.6X for domestic yaks and from 11.4 to 21.2X for wild yaks. The selection of these 16 samples was based on the availability of accompanying next-generation sequencing (NGS) data. The SRA format was converted into the FASTQ format using fastq-dump. Nanopore sequencing data were quality controlled using NanoFilt (parameters -q 10 -l 500). Low-quality and adapter sequences of NGS data were trimmed using fastp [27]. Samples with both nanopore and NGS sequencing data were assembled using wtdbg2 [28]. The assemblies were then polished using NextPolish [29]. Three cycles of nanopore sequencing data polishing and three cycles of NGS data polishing were performed.

### Genome annotation

First, the 16 assembled yak genomes were subjected to repeat sequence annotation using RepeatMasker [30]. De novo repeat sequence libraries for each yak genome were constructed using RepeatModeler [31] and then annotated using RepeatMasker. Tandem Repeats Finder [32] was used to annotate the tandem repeat sequences. Long terminal repeat libraries were constructed using LTR_retriever [33] and LTR_FINDER [34]. Additionally, RepeatProteinMask was used to annotate the genome with repetitive sequences. BosGru3.0 of the yak reference genome was used to train the species model of Augustus [35]. De novo gene structures were then predicted using Augustus. Furthermore, RNA-seq data from 156 samples (NCBI BioProject numbers PRJNA548123, PRJNA624986, PRJNA512958, PRJNA627310, PRJNA644042, PRJNA644608, PRJNA727968, and PRJNA822439) were retrieved for gene structure annotation. These transcriptome sequencing samples were derived from various organs and tissues of yaks, including the lung, biceps femoris, latissimus dorsi (LD), adjacent intermuscular adipose tissue (AA), cerebellum, cerebrum, heart, lungs, skin, and testis. Sequences that could be mapped to the genome were assembled using Trinity [36]. Finally, the gene structure annotation of each genome was performed using MAKER2 [37].

### Yak pangenome construction

The final pangenome was obtained through an iterative approach by comparing each genome with the reference genome or with the pangenome (reference and nonreference) obtained in the previous round. In the first round, we used the nucmer tool in the mummer package (with parameters -c 100 -fxdxb 500 -l 50) to compare one of the assembled genomes with the reference genome BosGru3.0 [38]. The results were filtered for one-to-one alignment, allowing rearrangements, using the delta-filter tool (with parameter -1) and further filtered using show-coords (with parameters -I 0.95 -L 100). To identify and extract the missing sequences of the reference genome in the assembled genomes, we utilized the get_absese_region.pl and get_seq.pl scripts in the ppsPCP package [39]. Furthermore, we used BLASTN to realign candidate nonreference sequences against the reference genome to filter out sequences that were redundant to the reference genome. Sequences with a regional similarity of more than 90% to the reference genome and a similarity rate exceeding 95% were removed.

The final nonreference sequences were then merged with the reference genome and used as the reference sequence for the next round. After 16 cycles, the final yak pangenome was obtained. For the nonreference sequences, genes exhibiting an overlap of 80% or more with the gene annotations of each genome were designated novel genes, following the criteria defined by Song et al. [40]. The gene sequences in the pangenome were compared with the NR and NT libraries of NCBI and Swiss-Prot for the functional annotation of genes. Additionally, the Gene Ontology (GO) and Kyoto Encyclopedia of Genes and Genomes (KEGG) annotations of the genes were obtained using ID associations between databases. Protein sequences of the gene transcripts were annotated using hmmscan for Pfam annotation.

### Gene PAV analysis

A total of 350 yak resequencing data (Additional file 1: Table S1) were collected from previous studies [3, 11, 12, 41–43], and low-quality and adapter sequences were removed using fastp for further analysis. The resequencing data were aligned to the yak pangenome using Bowtie2 [44]. To determine the presence of a gene, a defined threshold was established. This threshold stated that a gene would be considered present only if its exonic regions were covered by a minimum of two sequencing reads, with a cumulative read count exceeding 20% of the total length of the gene’s exonic regions. Therefore, the presence or absence of genes in the pangenome was determined using SGSGeneLoss v0.1 (minCov = 2, lostCutoff = 0.2) [23]. To conduct downstream analysis based on gene PAV information, missing genes were designated as ‘0’ and present genes as ‘1’, resulting in a binary gene PAV dataset. Principal component analysis (PCA) was performed on the binary gene PAV data using the vegan package in R software [45, 46]. Subsequently, a maximum-likelihood phylogenetic tree (1,000 bootstraps) was constructed based on the binary gene PAV data using IQ-TREE [47]. The population structure of yaks was analyzed using STRUCTURE based on PAV data [48].

To investigate gene PAVs under selection, we analyzed gene frequency differences between populations according to the population structure results mentioned above. Fisher’s exact test was used to detect the significance of gene frequency differences among the three populations. *P* values were corrected using the Benjamini‒Hochberg (BH) method. The thresholds for significance were set at a false discovery rate (FDR) < 0.001 and frequency differences > 2. Enriched GO terms and KEGG pathways were analyzed using the hypergeometric distribution test, with a threshold of FDR < 0.05.

Lan et al. [3] reported that some Jinchuan yaks had 15 rib bones. Therefore, trait information was obtained from that study. Gene PAV-GWAS was conducted using the PAVs of the shell genes as the genotype and employing the FarmCPU method [49, 50]. The significance threshold was set at 2.24 × 10^–5^ (0.05/2232).

### SNP identification and analysis

Data cleaning involved mapping clean data to the yak reference genome (BosGru3.0) using BWA v 0.7.17 [51]. GVCF files were generated using the HaplotypeCaller in GATK [52]. A total of 350 GVCF files were combined with CombineGVCFs and then processed by GenotypeGVCFs for genotype calling. SNPs were filtered using variantFiltration with the following parameters: –filter-expression ‘QD < 2.0 || FS > 60.0 || MQ < 40.0 || SOR > 3.0 || MQRankSum < −12.5 || ReadPosRankSum < −8.0’ –filter-name ‘snp_filter’ –genotype-filter-expression ‘DP < 2 || DP > 50’ –genotype-filter-name ‘dp_fail’. Finally, SNP loci with missing rates greater than 90% and minor allele frequencies (MAFs) < 5% were removed by VCFtools. The impact of SNPs was annotated using Variant Effect Predictor v99 [53]. Yak population structure was analyzed using ADMIXTURE [54], with K values ranging from 2 to 9 and a cross-validation set to the default human value of 5. Ten cycles were run for each K value. Gene flow among different yak populations was inferred using TreeMix [55] with the global parameter bootstrap 5,000.

### Mitochondrial genome assembly and analysis

The assembly of the mitochondrial genome was performed using GetOrganelle [56] based on yak resequencing data. Complete or near-complete sequences were aligned using MAFFT 7.0 [57]. A neighbor-joining tree was constructed using MEGA [58].

### RNA-seq data retrieval and expression analysis

RNA-seq data from various yak tissues, including the lungs, brain, cerebellum, latissimus dorsi, heart, biceps, external abdominal oblique, and testis, were retrieved from the NCBI by searching the ‘Organism’ for ‘*Bos grunniens*’ (NCBI BioProject numbers PRJNA548123, PRJNA624986, PRJNA512958, PRJNA627310, PRJNA644042, PRJNA644608, PRJNA727968, and PRJNA822439). A total of 156 samples were included, and these samples underwent quality control using the same methods as those used for the raw data processing described above. Clean reads were mapped to the genome using HISAT2 [59], and read counts for each gene were calculated using featureCounts [60]. FPKM values were used for gene expression calculations. We standardized the gene expression levels based on the total reads count per sample. Differential expression analysis was performed using DESeq2 [61]. The FDR, controlled by the BH method, was set at a threshold of less than 0.05, along with a fold change criterion exceeding 2. The tissue specificity index (TAU) was calculated using the following formula:

$$tau=\frac{n}{n-1}-\frac{{\sum }_{i=1}^{n}{x}_{i}}{\left(n-1\right)\times \underset{1\le i\le n}{{\text{max}}}\left({x}_{i}\right)}$$

In the formula, *n* denotes the number of groups, *x* denotes the mean value of gene expression in different groups and *i* denotes one of the groups. The indices range from 0 (broad expression) to 1 (restricted expression). For specifically expressed mRNAs and target genes of the specifically expressed lncRNAs, GO enrichment analysis was conducted using a hypergeometric distribution test. The GO annotations of all genes in the yak genome were used as the background.

### Multispecies mRNA and lncRNA analysis

In this study, RNA-seq data from yaks, cattle, high- and low-altitude pigs, chickens, goats, and sheep were collected [62]. Six tissues (heart, kidney, liver, lung, skeletal muscle, and spleen) from each species were sampled, and all the samples were untreated. The dataset comprises approximately 909 Gb of data, covering 30 individuals with a total of 180 samples (6 tissues per individual). After the clean data were mapped to the genome, the long noncoding RNA molecules of each species were identified using the CPC2, CNCI, and Pfam methods [63, 64]. The 1:1 orthologous genes between different species were identified using OrthoFinder [65]. The organ specificity of gene expression was calculated, and differential expression analysis was performed as described above for RNA analysis.

## Results

### Yak pangenome construction and gene PAV calling

**Fig. 1:**
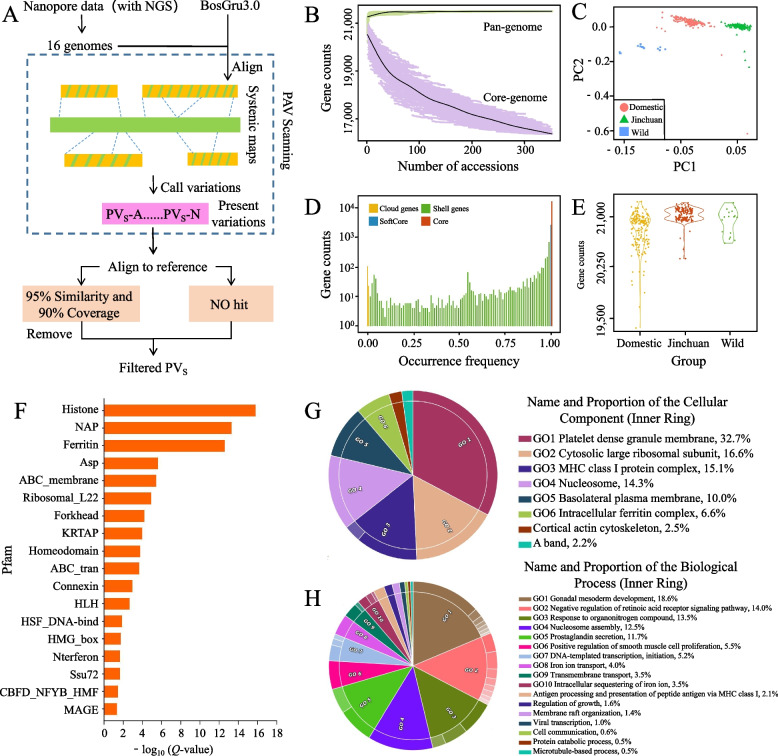
Yak pangenome construction. **A** System diagram of Yak pangenome construction based on third- and second-generation sequencing data. This is an iterative alignment process. Initially, 16 yak individuals, each possessing both third-generation and second-generation sequencing data, were selected for de novo assembly. Following independent assembly, the generated sequences were aligned against the reference genome (BosGru3.0). The purpose of this process is indicated by the dashed box, which involves extracting nonreference sequences from the 16 genomes through homology-based alignments with the reference genome. This process is iterative, meaning that the nonreference sequences from the previous round are combined with the reference to form the ‘reference’ for the next round, thereby eliminating redundant sequences among the 16 genomes. Finally, all nonreference sequences were aligned against the reference genome again to further remove redundancy. **B** The fitted curve shows an increase in the total number of genes and a decrease in the number of core genes in the yak pangenome as individuals increase. Each given number of sample combinations was randomly selected 10,000 times. The upper and lower boundaries of the pink and green regions correspond to the maximum and minimum numbers of genes, respectively. **C** PCA based on binary gene PAV data. **D** Distribution of the number of core, softcore, shell and cloud genes. **E** Distribution of genes in Jinchuan, other domestic and wild yak populations. **F** Pfam enrichment analysis results of shell genes. **G–H** Shell genes were enriched (*P* < 0.05) in some GO terms belonging to cellular component and biological process

Nanopore and NGS genome data from 16 yak individuals were subjected to de novo assembly and polishing to produce 16 yak genomes ranging from 2.63 to 2.73 Gb (Table S2). The number of genes predicted in the 16 genomes ranged from 19,858 to 21,238 (Table S3). To construct the pangenome of yaks, we employed an iterative comparison strategy by aligning these 16 genomes with a reference genome (Fig. 1A). This approach aimed to identify sequences that were missing in the reference genome. We identified 290 Mb of nonreference sequences. Consistent with the threshold chosen by Song et al. [40], genes from the 16 genomes that exhibited 80% overlap with these nonreference sequences were classified as PAV-related genes. Using these criteria, we identified a total of 504 new genes.

The “Methods” section states that resequencing data of 350 samples from multiple previous studies on yaks (Table S1) were collected. These samples were obtained from 51 yak breeds, and they included genomic data from yaks residing in various regions. After mapping the resequencing data of 350 samples to the yak pangenome, 16,330 (75.69%), 2,614 (12.12%), 2,506 (11.62%), and 125 (0.57%) core, softcore, shell, and cloud genes, respectively, were identified (Fig. 1D). The core gene set consisted of genes detected across all accessions, whereas the softcore gene set included genes found in more than 99% of the accessions. The shell genes were identified in more than 1% but less than 99% of the accessions, whereas the cloud genes were observed in fewer than 1% of the accessions [23]. The softcore, shell, and cloud genes represented variable genes, collectively accounting for 24.3% of the total gene count in the yak pangenome. This indicated that most of the genes did not experience loss of mutations during domestication and improvement.

To investigate the association between the number of genes in the yak pangenome and sample size, random sampling was conducted. The results demonstrated that the number of genes in the pangenome approached saturation as the sample size increased to approximately 100 (Fig. 1B). However, the number of core genes in the yak population continued to decrease with an increasing number of samples, suggesting that the number of core genes in the population would have continued to decrease if more yak samples had been collected for sequencing. This finding indicated that different yak varieties in various regions underwent abundant gene PAV selection during the domestication process.

Binary gene PAV-based PCA revealed that yaks could be categorized into three distinct groups: wild, domestic, and Jinchuan (Fig. 1C), which aligned with the SNP-based study conducted by Lan et al. [3]. Notably, Jinchuan yaks constitute a type of domestic yak with exceptional characteristics. Specifically, approximately 52% of Jinchuan yaks possess 15 pairs of ribs, in contrast to the typical 14 pairs found in other yaks. Moreover, Jinchuan yaks exhibit numerous advantages, such as strong resilience, high meat and milk production, and robust reproductive capabilities [3]. This finding indicated that the polymorphism information of the PAV gene is population-specific and can aid in distinguishing between different populations, as demonstrated by SNP-based population PCA.

The analysis of gene PAVs revealed that the number of genes varied among the different yak populations (Fig. 1E). In domesticated yaks, the number of genes ranged from 19,386 to 21,242, demonstrating extensive variations attributable to PAVs within individual genomes. This study included a total of 51 yak breeds, with significant differences not only in morphology but also in terms of altitude and environment. For instance, of the 174 domesticated yak samples, 118 belonged to the Tibetan Plateau type, while 24 belonged to the Hengduan Mountains type. The three yak breeds with the fewest genes were Cuona yak (19,386 genes), Cuona yak (19,695 genes), and Dingqing yak (19,905 genes), whereas Jinchuan yak (21,224 genes), Tianzhu yak (21,238 genes), and Jiangda yak (21,242 genes) had the highest gene counts. The existence of PAVs and the resulting variations in gene count are associated with selection processes, genetic drift, and inbreeding in domesticated yak populations. These structural variations primarily occur in shell genes, and GO analysis revealed enrichment in functions such as positive regulation of smooth muscle cell proliferation, regulation of growth, and the MHC class I protein complex (Fig. 1G and H), which are closely related to yak growth and immunity. Additionally, Pfam enrichment analysis of shell genes revealed interesting domains (Fig. 1F), such as keratin-associated proteins, which have implications for traits such as hair in animals [66] and may be connected to the distinct hair characteristics observed in yaks.

### Selection signal analysis

**Fig. 2:**
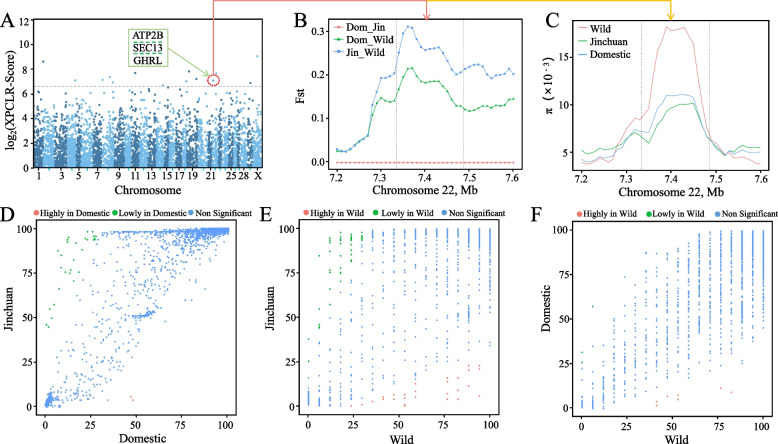
Selection analysis of yak populations. **A** Whole genome-wide selection signal analysis by XP-CLR. *F*_st_ analysis results (**B**) and pi value distribution (**C**) for ATP2B, SEC13 and CHRL, as well as 4 kb upstream and downstream of genes in the three populations. Gene frequency analysis results for the three combinations: domestic vs. Jinchuan (**D**), Jinchuan vs. wild (**E**) and domestic vs. wild (**F**)

A genome-wide scan of SNP information among populations was conducted using XP-CLR with a window size of 100,000 bp. A total of 17 regions with selection signals were identified (Fig. 2A), and 23 genes were found within these regions. These findings demonstrate that a larger population size allows for the detection of more selection signals. Among the genes with selection signals, *ATP2B*, *SEC13*, and *CHRL* were found to be associated with growth and development traits. *F*_st_ analysis of these three genes in wild, Jinchuan, and other domesticated yaks revealed no genetic differences between Jinchuan and other domesticated yaks, but significant genetic differences were observed between Jinchuan and wild yaks (Fig. 2B). These results may be attributed to the fact that wild yaks exhibit larger body sizes than domesticated yaks. Analysis of the pi values in each population revealed that the three genes exhibited greater nucleic acid diversity in wild yaks than in other yaks (Fig. 2C). Gene frequencies varied among populations; therefore, we utilized Fisher’s exact test to identify gene PAVs under selection in the populations. The Jinchuan yak population is a distinct population of domestic yaks. Analysis of gene frequency revealed 48 significantly more genes and 32 lower-frequency genes in Jinchuan yak than in the wild yak population (Fig. 2E, Table S4). Notably, BosGru3G019931 (transcription factor SOX-3) and BosGru3G006135 (transcription factor jun-D) exhibited low gene frequencies in wild yaks. On the other hand, three genes had higher gene frequencies in other domestic yaks than in wild yaks, while seven genes had lower frequencies (Fig. 2F, Table S5). Several genes displayed significant frequency differences. For instance, the novel gene Novel_gene256 (multidrug resistance-associated protein 4-like protein) was entirely absent in wild yaks, which may be relevant to the domestication of domestic yaks. However, it is worth mentioning that this phenomenon could also be attributed to other factors, such as the challenges associated with obtaining wild yaks, resulting in limited genomic data from wild yaks and potential failure to detect certain wild yaks carrying this gene. Furthermore, in the comparison of gene frequencies between Jinchuan and other domestic yaks, it was found that Jinchuan possessed 30 high-frequency genes, whereas other domestic yaks had only two high-frequency genes (Fig. 2D, Table S6). These findings indicate that domestic yaks lack a consistent PAV gene due to breeding objectives and environmental factors, resulting in fewer high-frequency genes in these yaks than in Jinchuan and wild yaks. Comparative analysis revealed that Jinchuan yaks possessed certain unique genes during the domestication process, such as BosGru3G016459 (G-protein coupled receptor 27), which could be associated with neurotransmitter concentration [67]. The identification of genes under selection through SNPs and gene PAV analysis provides valuable insights into the domestication and breeding of yaks based on gene variation and function.

### Breed-specific genes in the yak

Previous analyses have indicated that various domesticated yak varieties may possess numerous inconsistent PAVs. These PAVs could arise through gene PAV selection, genetic drift, and inbreeding, leading to the acquisition of new genes by different varieties. Some of these genes may be responsible for variety-specific traits, and identifying them could enhance our understanding of the yak population genome. When yaks from different varieties crossbreed, their offspring may inherit all the genes from both varieties. Furthermore, specific genes found in one parent may only appear on one of the chromosomes of the offspring genome. Hence, there is the potential for gene PAVs to exist between the two haplotypes.

**Fig. 3:**
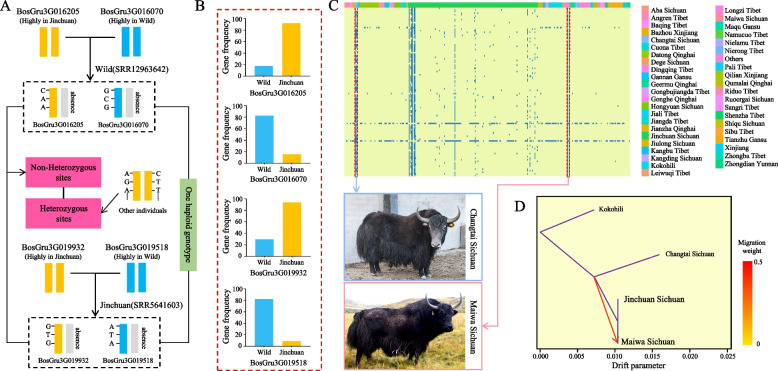
Variety-specific genes in yak populations. Cross-population hybridization may occur between wild and Jinchuan yaks. **A** BosGru3G016205 and BosGru3G016070 are homozygous in one wild population but show widespread heterozygosity in their respective higher-frequency populations. BosGru3G019932 and BosGru3G019518 are homozygous in another Jinchuan yak individual but show widespread heterozygosity in their respective higher-frequency populations.** B** Gene frequencies of four genes with frequency differences in the Jinchuan and wild populations. **C** Gene PAV heatmap showing the Maiwa- and Changtai-specific genes. **D** Gene flow between Changtai and Maiwa yaks

Based on the SNP and gene PAV data, we detected genes that exhibited significant frequency variations across populations but were present within individual yaks. For instance, in a Jinchuan yak (NCBI, number SRR5641603), both the BosGru3G019932 and BosGru3G019518 genes were identified (Fig. 3A). Notably, these genes exhibited significant differences in frequency between the wild and Jinchuan yak populations (Fig. 3B). In this particular Jinchuan individual, both genes were found to be homozygous, whereas in other Jinchuan individuals without both genes, BosGru3G019932 was heterozygous, and BosGru3G019518 was heterozygous in other wild individuals not possessing both genes. These findings suggest that hybridization between Jinchuan and wild yaks potentially facilitated the exchange of variety-specific genes between the populations. Similarly, a wild yak individual (NCBI, number SRR12963642) harbored the BosGru3G016205 (highly prevalent in Jinchuan) and BosGru3G016070 (highly prevalent in the wild) genes. Both of these genes were observed to be homozygous and widely heterozygous in their respective high-frequency populations. The detected genotypic patterns of these genes may serve as a model depicting how two individuals possessing variety-specific genes can acquire all the genes from their parents through hybridization, potentially resulting in gene PAVs between the two haplotypes. By combining the gene PAV data, along with information on the heterozygous and homozygous states of individual genes, it is possible to infer hybridization processes among yak varieties. This methodology could greatly facilitate the study of yak varieties, particularly the formation of hybrid varieties, and even aid in the evaluation of yak germplasm resources.

In the present study, a total of 51 yak breeds were collected to explore breed-specific genes. To accurately assess yak variety-specific genes, we retained only 43 yak varieties with at least three individuals. The analysis of genes in each breed revealed that there were 228 specific genes in the reference genomes of the Maiwa and Changtai yaks. Through a comparison with genes in the NT database, it was found that 121 genes originated from sequencing of *Theileria parva*. After removing these genes, a total of 107 genes specific to Maiwa and Changtai yaks were obtained (Fig. 3C, Table S7). Gene flow analysis revealed gene flow between Maiwa and Changtai (Fig. 3D), indicating a close relationship between these two yak varieties. Similarly, gene flow was observed between cattle and Jinchuan yaks (Fig. S1). The discovery of variety-specific genes provides valuable information for variety identification and breeding. The findings of this study highlight the effectiveness of gene PAV analysis in mining variety-specific genes.

### Gene PAV-GWAS and gene-CDS-haplotype analysis

**Fig. 4:**
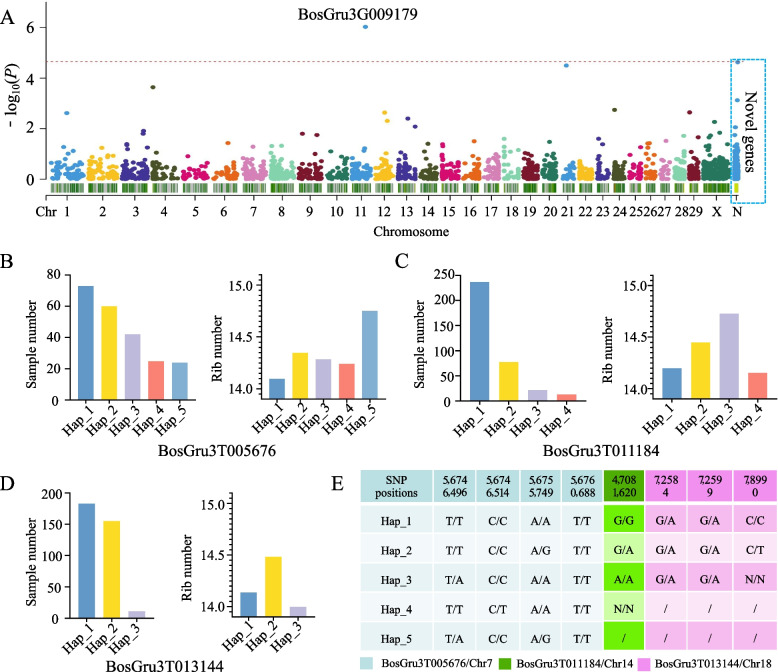
PAV-GWAS and gene-CDS haplotypes analysis of rib quantity traits in yak populations. **A** Results of gene PAV-GWAS analysis of yak rib number. **B** Sample numbers of five gene-CDS haplotypes and corresponding mean rib numbers for BosGru3T005676; **C** sample numbers of four gene-CDS haplotypes and corresponding mean rib numbers for BosGru3T011184; **D** sample numbers of three gene-CDS haplotypes of BosGru3T013144 and the corresponding mean numbers of ribs; **E** Nucleotide information of various gene-CDS haplotypes

Gene PAV-GWAS and gene-CDS-haplotype analysis were subsequently conducted to investigate the impact of the PAV and SNP genes on traits in yak populations. GWAS analysis focused on the rib number phenotype in yaks, with gene PAVs serving as markers. The analysis revealed that the BosGru3G009179 (phosphatidylinositol N-acetylglucosaminyltransferase subunit H) gene on chromosome 11 was significantly associated with the number of ribs in yaks (Fig. 4A). Notably, this gene was not identified through SNP-GWAS analysis [15], highlighting the potential role of the PAV gene in regulating traits.

Given the large number of genes associated with rib counts obtained through SNP-GWAS analysis, our ability to narrow the list of candidate genes, such as TUBA8 and CDC42EP1, was limited [3]. It is worth noting that certain traits may have preferences for specific gene-CDS haplotypes [68]. Therefore, conducting a genome-wide gene-CDS haplotype analysis may reveal that certain genes are associated with specific haplotypes related to yak rib counts.

For instance, Hap_1 and Hap_5 in BosGru3T005676 were present in 73 and 24 samples, respectively (Fig. 4B). The mean number of ribs was 14.1 for samples with Hap_1 and 14.75 for samples with the Hap_5 haplotype. Additionally, four other genes, such as BosGru3T011184, exhibited four haplotypes in 237, 78, 22, and 13 samples (Fig. 4C). The average number of ribs for the sample with Hap_3 was 14.73, while the samples with Hap_1 and Hap_4 had average rib counts of 14.2 and 14.15, respectively. Furthermore, three gene-CDS haplotypes of the BosGru3T013144 gene exhibited phenotypic differences (Fig. 4D).

A gene haplotype refers to a combination of variant sites on a single gene that is typically associated with a specific trait. These gene haplotypes play a crucial role in identifying variations in the affected trait within the gene region (Fig. 4E). By utilizing gene PAV-GWAS and gene-CDS-haplotype analysis, we have gained valuable insights into genes that may be potentially associated with yak rib count.

### Population structure analysis

**Fig. 5:**
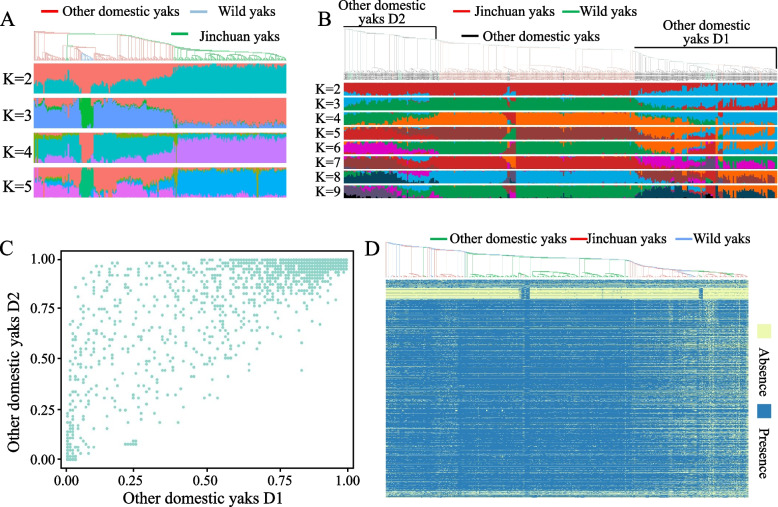
Phylogenetic tree of yaks and population structure obtained based on SNPs (**A**) with binary PAV data (**B**). **C** Scatter plot of gene frequencies between clade D1 and clade D2 of other domesticated yaks. **D** Heatmap of gene PAVs constructed in the order of the phylogenetic tree

SNP-based and gene PAV-based phylogenetic trees can facilitate a clear distinction between domesticated yaks and Jinchuan yaks, highlighting the accuracy of gene PAV polymorphism analysis in yaks. According to the SNP-based population structure analysis, when K = 3, wild yaks were found to be completely independent. Some Jinchuan yaks and other domesticated yaks were related to each other (Fig. 5A). However, the gene PAV-based tree provides more detailed clustering information and population structure information. This clade divides the domesticated yak into two parts, namely, clade D1 and clade D2 (Fig. 5B). Notably, in the population structure analysis based on the PAV gene, wild yaks were mixed with Jinchuan yaks and other domesticated yaks, indicating that SNP and gene PAV polymorphisms in individuals can reveal distinct population structures. The population structure analysis at K = 3 (Fig. S2) demonstrated that the domesticated yaks in Clade D2 were more closely related to the Jinchuan yaks. Moreover, the scatter plots of gene frequencies for Clade-D1 and Clade-D2 revealed that a large number of genes had higher frequencies in Clade-D2 than in Clade-D1 (Fig. 5C). Additionally, the gene PAV heatmap, constructed based on the associations in the phylogenetic tree, showed that many neighboring yaks possess the same specific genes (Fig. 5D). However, these genes are not breed-specific genes and may be associated with hybridization between yak breeds.

### Gene expression atlas of multiple organs and tissues of the yak

**Fig. 6:**
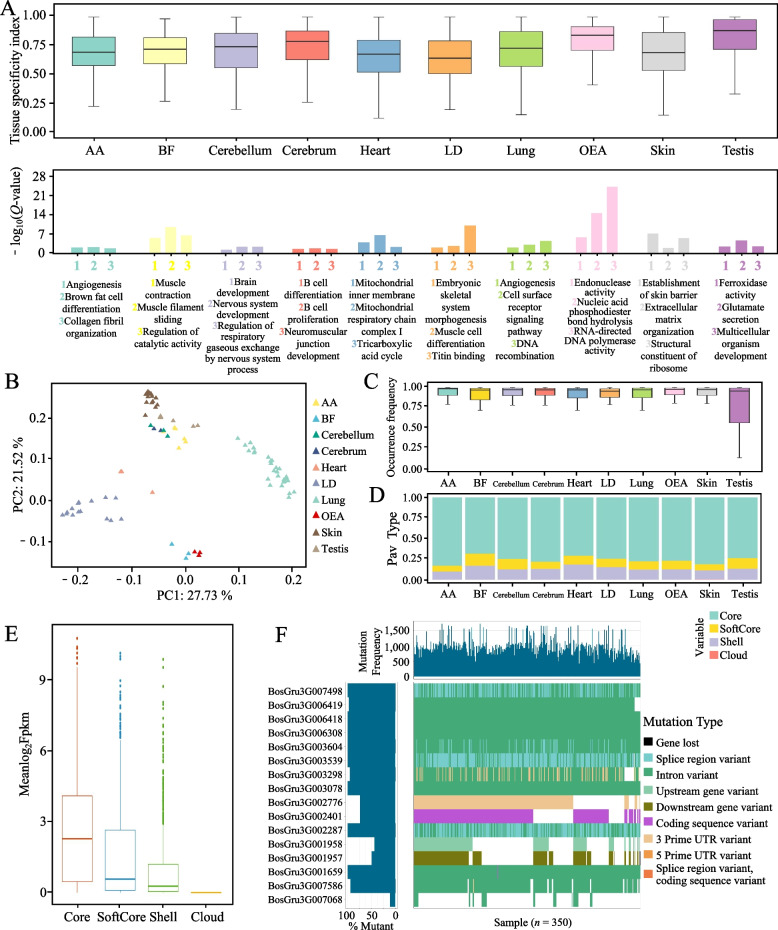
Multiorgan and tissue expression profiles and population variation in yaks. **A** Genes specifically expressed in each organ or tissue and their specificity indices. Biceps femoris (BF), external abdominal oblique (OEA), yak latissimus dorsi (LD), adjacent intermuscular adipose tissue (AA). The bar plot under the box plot shows the terms of organ-specific genes enriched in the biological process. **B** PCA based on the expression values of all the samples. Gene frequencies of genes specifically expressed in each organ in yaks (**C**) and a stacking map of gene types (**D**). **E** Distribution of expression values of core, softcore, shell and cloud genes in all samples. **F** Waterfall map of gene load in yak population for differentially expressed genes living between high- and low-altitude yaks

Transcriptome analysis is a crucial technology utilized in the study of animal growth, development, and environmental adaptation. This particular study focused on collecting transcriptomes from various yak organs and tissues, including the lung, biceps femoris, yak LD and its AA, cerebellum, cerebrum, heart, lungs, skin, and testis. The analysis revealed that core genes exhibited the highest expression levels across all samples, while genes with lower frequencies displayed lower expression levels (Fig. 6E). Highly conserved genes play a fundamental and significant role in the various life activities of yaks. Although variable genes exhibited low expression levels, their importance cannot be overlooked, as they potentially contribute to the development of diverse phenotypes. PCA demonstrated the specificity of gene expression in different organs and tissues (Fig. 6B). By calculating the TAU values, we identified genes that were specifically expressed in all organs and tissues. The testis exhibited the highest specificity index, whereas the yak dorsalis muscle displayed the lowest specificity index for gene expression (Fig. 6A). These specifically expressed genes are enriched in various functions, such as muscle cell differentiation, which is particularly enriched in genes specifically expressed in LDs. The gene PAV analysis illustrated that genes specifically expressed in different organs exhibited varying levels of conservation. The biceps femoris had the highest percentage of genes with a shell structure, while the AA had the lowest percentage of these genes (Fig. 6D). However, the distribution of gene frequencies did not align with the proportion of shell genes (Fig. 6C). For instance, genes specifically expressed in the testis had the highest median gene frequency, despite not having the smallest proportion of shell genes.

The lungs of yaks are vital organs that have the ability to adapt to the challenging environment of the plateau [69, 70]. This study investigated the differences in gene expression between yaks living at different altitudes, such as the Tibetan Plateau type and the Hengduan Mountains type. A total of 51 genes were found to be differentially expressed in yaks at altitudes of 3,400 and 5,000 m. Mutational load analysis of these genes revealed a diverse range of mutations and mutation types (Fig. 6E). Specifically, the BosGru3G006130 gene showed little variation in the population, suggesting that it may not play a role in the adaptation of yaks to the plateau. On the other hand, genes such as BosGru3G020612 had lost gene variants in multiple populations, while BosGru3G006132 and BosGru3G006131 had numerous variants upstream of the gene. Further research is needed to determine whether these variants directly cause gene loss or affect gene expression. Importantly, several splice region variants were discovered in the BosGru3G007895 gene that can lead to changes in gene splicing and potentially impact phenotypic traits. In conclusion, these findings provide valuable insights into the genetic variations within yak populations.

### Gene and lncRNA expression analysis between high- and low-altitude regions

**Fig. 7:**
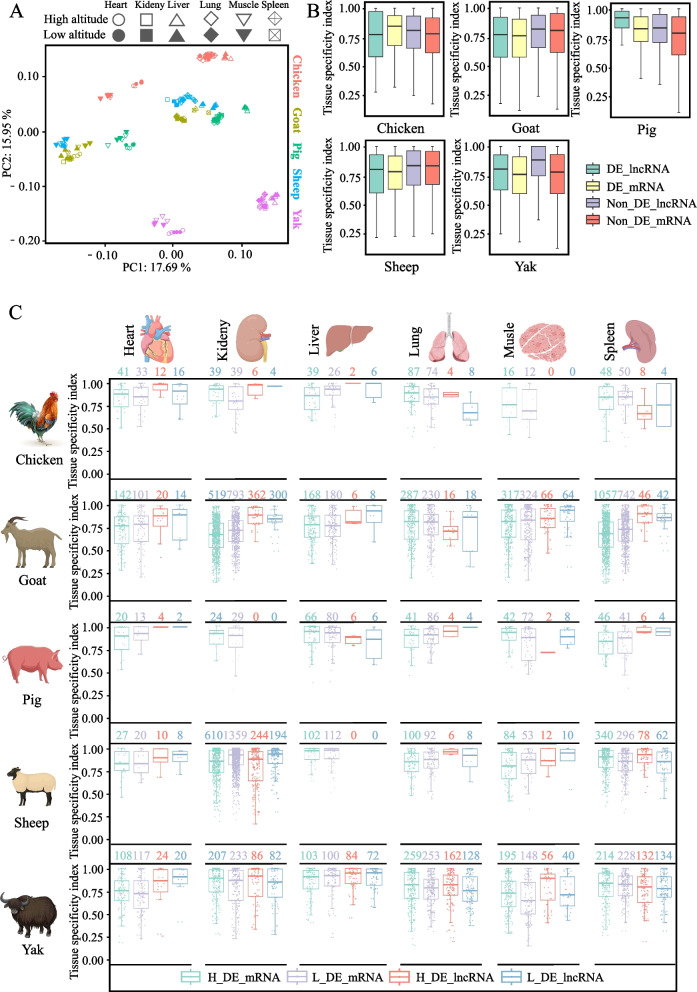
Expression analysis of genes and lncRNAs between high- and low-altitude individuals of multiple species. **A** PCA of gene expression based on 1:1 orthologs between multiple species. **B** Expression-specific index distribution of differentially expressed mRNAs and lncRNAs in six organs between high- and low-altitude individuals. **C** Expression specificity index of differentially expressed mRNAs and lncRNAs in each organ between high- and low-altitude individuals of each species

Although yak is the most well-known species living in the highlands, other animals, such as Tibetan pigs, Tibetan sheep, and Tibetan chickens, also inhabit the QTP. However, it remains unclear whether these animals utilize similar or different mechanisms to adapt to the plateau. To address this question, RNA-seq data from high- and low-altitude varieties of yaks, cattle, sheep, goats, chickens, and pigs were analyzed. PCA revealed that samples from each species formed distinct clusters, indicating species and organ specificity in gene expression (Fig. 7A). Notably, liver samples tended to cluster on the right side of the PCA map, while muscle samples clustered on the left side. This highlights the pronounced differences in gene expression between species and organs.

A study conducted by Tang et al. [62] examined the mRNA expression of various species. In our study, we identified and analyzed not only lncRNA molecules but also mRNAs from the RNA-seq data of each species. The yak analysis utilized 180 samples from multiple organs, resulting in the identification of 29,167 lncRNA molecules. Additionally, we identified 10,926, 11,882, 15,586, and 10,030 lncRNA molecules in pigs, sheep, goats, and chickens, respectively. We used the same methods and thresholds for the analysis of differentially expressed lncRNAs and mRNAs in both high- and low-altitude animals (Table S8). Pearson correlation coefficients were calculated to determine potential regulatory associations between lncRNAs and mRNAs (Table S9).

The evaluation of organ expression specificity for differentially expressed and non-differentially expressed genes or lncRNAs (coexpressed with DE mRNAs) revealed (Fig. 7B) that non-differentially expressed lncRNAs exhibited the highest organ expression specificity in yaks, while this pattern was not consistent in other animals. For instance, in chickens, the organ expression specificity of differentially expressed mRNAs was the highest. These findings suggested that species may exhibit different patterns of gene expression or regulation to adapt to high altitudes.

By analyzing the organ expression specificity of DE mRNAs versus lncRNAs in animals at high and low altitudes (Fig. 7C), we observed variations in the number of organ-specific expressed genes among different species. The differences in gene expression and the specificity of lncRNAs between yak and cattle vary across six organs. It is worth noting that the number of mRNAs specifically highly expressed in the heart and lungs of yaks (108 and 259, respectively) is significantly higher than in the other three species, excluding goats (142 and 287). This implies that the hearts and lungs of yaks and high-altitude goats are relatively important during high-altitude adaptation. Meanwhile, as a type of non-coding RNA regulating coding genes, the expression patterns of lncRNAs represent potential regulatory mechanisms. The number of differentially expressed mRNAs with lung-specific expression in yaks is similar to that in high-altitude goats, but the number of lung-specifically expressed lncRNAs in yaks (162) is higher than in high-altitude goats (16), indicating the potential importance of lncRNAs in the high-altitude adaptation of yaks.

## Discussion

**Fig. 8:**
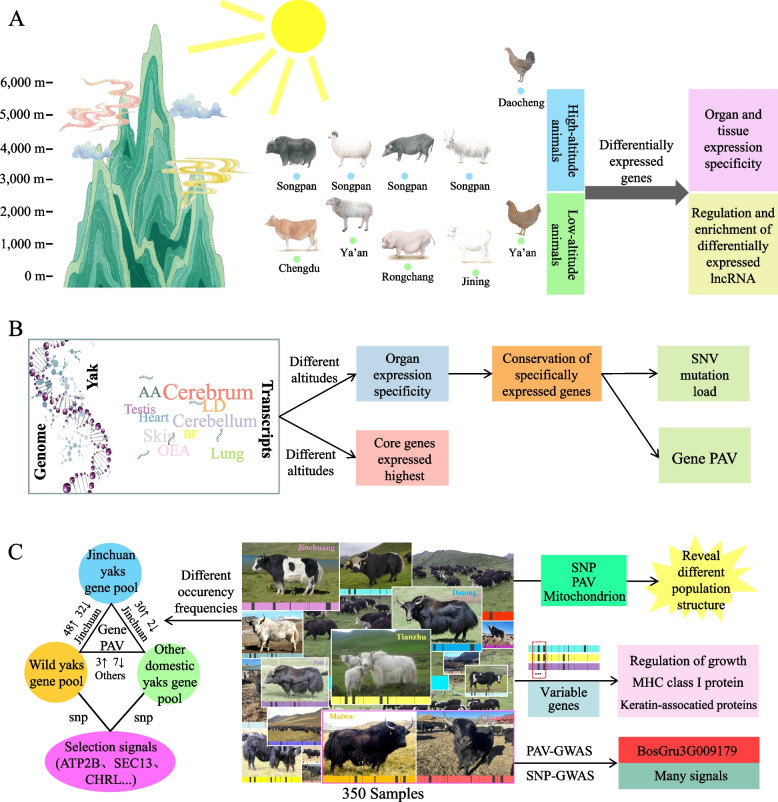
Summary of pangenome and transcriptome studies in the present study. **A** The present study explored the differential expression of lncRNAs across multiple species by comparing high-altitude and low-altitude breeds. The findings reveal the widespread regulatory role of lncRNAs in highland adaptation, as well as their species specificity. **B** The present study integrated the tissue-specific expression patterns of yak genes with population variation burden (including PAV and SNP), uncovering potential selection patterns of functional genes. **C** Through comprehensive analysis of the yak population genome, particularly via pan-genome analysis, the present study revealed more population-specific genes and candidate functional genes associated with specific traits

During the long period of domestication and breeding, various variants were under selection pressure in different directions. In recent years, numerous studies have utilized genomic or transcriptomic approaches to investigate important traits and identify candidate genes [71–73]. However, these studies primarily focused on the identification of DEGs or SNP loci based on a reference genome. Additionally, it is important to note that gene absence variants identified using SGSGeneLoss software do not necessarily indicate a complete loss of the gene but rather the likelihood that a portion of the gene has been lost [74], resulting in the loss or alteration of gene function. For instance, in the case of tomatoes, some promoter sequences of flavor-related genes were lost during the breeding process to prioritize yield or resistance breeding objectives [23]. Consequently, the retrieval of lost genes represents an effective approach for yak breeding. To accomplish this goal, it is necessary to detect genes from a wider range of yak varieties and design appropriate crossbreeding strategies. For this purpose, we constructed a yak pangenome, capturing 290 Mb of nonreference contigs and identifying 504 novel genes. This pangenome serves as a valuable resource for functional genomics studies in yaks. Notably, our research revealed significant genetic variation among different yak varieties, providing a foundation for the discovery and utilization of functional genes. For example, we found that the pangenome contained enriched shell genes associated with the regulation of growth, the MHC class I protein complex, and gene families involved in hair development (Pfam enrichment analysis). This result suggested that the process of artificial selection during domestication for specific traits can lead to the loss of genes associated with other traits in the yak genome (Fig. 8C).

A more consistent genetic background enhances the ability to identify specific genes, resulting in the identification of numerous genes with varying frequencies in domestic yaks (including Jinchuan) and wild yaks. Among these genes, we discovered interesting genes, such as Novel_gene 239, which is significantly more prevalent in wild yaks than in domestic yaks. This gene is an adiponutrin-like gene associated with obesity and fat accumulation in animals [66, 75]. Additionally, we found a highly prevalent antiviral immunity-related gene [67], BosGru3G002943, in wild yaks. On the other hand, the multidrug resistance-associated protein 4-like gene was found at higher frequencies in domestic yaks (including Jinchuan yaks) but was completely absent in the wild yak population. We also identified a new gene, Novel_gene320, with a high frequency in Jinchuan yaks. This discovery indicates that the frequencies of various genes differ between wild and domestic yaks due to human domestication. Importantly, the identification of these novel genes from nonreference genomes highlights the importance of constructing a yak pangenome to further study the effects of domestication on the yak genome. Furthermore, we observed certain genes that exhibited significant differences in frequency among populations while coexisting within an individual. For instance, a specific Jinchuan yak possessed the genes BosGru3G019932 and BosGru3G019518, which showed substantial frequency differences between the wild and Jinchuan yak populations. Notably, in this individual, both genes were found to be homozygous, whereas BosGru3G019932 showed heterozygosity in other Jinchuan individuals, and BosGru3G019518 demonstrated heterozygosity in other wild individuals. These findings suggest a process of hybridization, wherein breed-specific genes are exchanged between Jinchuan and wild yaks. Therefore, we propose a ‘two-haplotype genomic hybridization model’ (Fig. 3A) to elucidate the hybridization patterns among breeds based on the frequency and heterozygosity results of genes in different populations, as well as the fusion of PAV information. This method greatly facilitates the study of interspecific hybridization in yaks and the evaluation of germplasm resources.

A substantial number of DEGs contribute to the understanding of potential mechanisms involved in plateau adaptation. However, a deeper understanding of these genes through multidimensional analysis is necessary. Genome variants can influence the phenotype by altering the protein sequence or regulating gene expression. Therefore, it is crucial to analyze the mutation load of DEGs to investigate selection pressure [68] (Fig. 8B). In this study, an integrated transcriptome analysis combined with PAV and SNP analysis was conducted according to the pangenome. Expression-specific index calculations and functional analyses of multiple organs and tissues revealed genes associated with organ function. For instance, genes (BosGru3G001947, BosGru3G002839, BosGru3G006310, etc.) involved in the mitochondrial inner membrane and mitochondrial respiratory chain complex I, which are specifically expressed in the yak heart, are associated with heart function [69, 70]. Organs and tissues such as the heart, AA, cerebrum, LD, and skin have only small percentages of genes that are specifically expressed as shell genes. This indicates that the functions of these organs and tissues were not under stronger selection of the PAV gene during domestication. On the other hand, the genes specifically expressed in the BF, which had the greatest proportion of shell genes, were enriched in GO terms such as muscle contraction and muscle filament sliding. This may be related to the selection for muscle-related traits during domestication. Conducting functional studies on these shell genes will aid in the identification of additional breeding-related genes.

A plethora of lncRNAs have been identified in mammalian research, inclusive of human studies [76, 77]. Nevertheless, the functional characteristics and roles of a significant portion of these lncRNAs remain elusive [78]. To investigate the functions of lncRNAs, it is necessary to identify potential functional lncRNAs through various methods. Previous studies have focused mainly on the possible involvement of lncRNAs in the regulation of plateau adaptation-related genes in yaks [18, 79]. But they were limited to certain tissues or organs, such as the brain, cerebellum and heart. Whereas, this study was conducted on 6 different organs. First, the present study revealed the prevalence of this phenomenon through DE lncRNA studies in multiple species (Fig. 8A). This revealed that lncRNAs have widespread expression differences among high-altitude individuals of various species. Furthermore, we identified DE lnRNAs that potentially regulate DE mRNAs and are specifically expressed in the lung and spleen. We know that the patterns of lncRNA regulation on target genes are diverse, but calculations based on correlation coefficients can rapidly identify potential lncRNA-gene regulatory relationships. Therefore, the abundant and specific expression of DE lncRNAs in the lung and spleen of yak provides a resource for further investigating the mechanisms behind mRNA expression changes. It provides new information on the transcriptional regulation of highland adaptation in yaks.

An intriguing discovery in this study was the identification of 107 unique genes in Changtai and Maiwa yaks, which are located on the contigs of the reference genome. These genes are often overlooked during SNP analysis. Additionally, contrary to previous findings regarding yak population structure, the results of the PCA in this study, using binary gene PAV data, revealed the classification of yaks into three distinct groups: wild, domestic, and Jinchuan. The polymorphism information of the gene PAV proved to be useful in distinguishing different populations and contributed to SNP-based population PCA. Moreover, PAV-based population structure analysis serves as a valuable supplement to SNP-based analysis, enabling a more detailed examination of population structure [23, 80]. For example, although wild yaks were clustered together in the SNP-based phylogenetic tree, their positions in the tree constructed using gene PAV data were more dispersed. This can be attributed to genetic exchange resulting from interbreeding between wild and domestic yaks during grazing. Therefore, utilizing different methods in the analysis of population structure enhances our understanding of yak kinship.

The Jinchuan yak is a remarkably distinct yak subtype, with 52% of individuals possessing an extra pair of ribs, resulting in a total of 15 pairs [3]. In this study, the BosGru3G009179 gene exhibited a significant association with the number of ribs in yaks when gene PAVs were utilized as markers. Several genes analogous to BosGru3G009179 are known to play a role in bone-related traits in animals, such as joint curvature in Belgian blue cattle caused by abnormal gene splicing [81] and cannon bone circumference in sheep [82]. Consequently, it is plausible that BosGru3G009179 is involved in the regulation of rib development through unfamiliar regulatory pathways. With the advancement of sequencing technologies, third-generation sequencing data with deeper sequencing depth and higher sequencing quality can now be obtained at acceptable costs, greatly enhancing the quality of analysis. The depth of data utilized in this study is not yet optimal, implying that higher quality sequencing data analysis may uncover additional novel genes. Furthermore, conducting various animal model experiments based on complex analysis results will further contribute to the understanding of molecular mechanisms.

## Conclusions

In conclusion, the main ideas and notable findings of this study are summarized in Fig. 8, which combines genomic and transcriptomic data from yaks to provide a comprehensive analysis of compelling issues. This study contributes new insights and resources concerning the domestication process, variety specificity, plateau adaptation, and other crucial aspects for future yak research.

### Supplementary Information

**Additional file 1: Table S1.** Information of 350 yak resequencing samples.

**Additional file 2: Table S2.** Genome sizes of 16 assembled yak genomes.

**Additional file 3: Table S3.** Gene numbers in 16 assembled yak genomes.

**Additional file 4: Table S4.** Genes had significant different frequencies between Jinchuan and wild yaks.

**Additional file 5: Table S5.** Genes had significant different frequencies between wild and other domestic yaks.

**Additional file 6: Table S6.** Genes had significant different frequencies between Jinchuan and other domestic yaks.

**Additional file 7: Table S7.** The information of 107 genes specific to Maiwa and Changtai yaks.

**Additional file 8: Table S8.** Differentially expressed lncRNA and mRNAs between yak and cattle.

**Additional file 9: Table S9.** Potential regulatory relationships between lncRNA and mRNAs in yak.

**Additional file 10: Table S10.** SNPs come with LD with flanking gene PAVs.

**Additional file 11: Fig. S1.** The gene flow between yaks and cattle.

**Additional file 12: Fig. S2. A** Cross-validation error curves to divide up training data into k-folds; **B** Phylogenetic tree of yaks constructed based on their mitochondrial genomes.

**Additional file 13: Fig. S3.** The log_2 _(fold change) values of differentially expressed lncRNA and mRNAs between high and low altitude animals.

**Additional file 14: Fig. S4. A** Electrophoresis of the PCR products of BosGru3G016459, BosGru3G006135, BosGru3G002214, Novel_gene256, Novel_gene248, BosGru3G010044, Novel_gene109, BosGru3G019931, and BosGru3G009179. **B**–**E** represent heatmaps for BosGru3G016459, BosGru3G006135, BosGru3G002214 in Jinchuan and wild yaks, Novel_gene256 and Novel_gene248 in domestic and wild yaks, BosGru3G019931, BosGru3G010044, Novel_gene109 in Jinchuan and wild yaks, BosGru3G009179 in yaks with 15 pairs of ribs and 14 pairs of ribs, respectively. Yellow indicates the presence of PCR products of corresponding length for the gene in the individual, while blue indicates the absence of PCR products of corresponding length for the gene in the individual.

## Data Availability

The yak pangenome assembly and annotation files and scripts are available at Figshare database (https://figshare.com/articles/dataset/yak_pangenome/22292737).

## Abbreviations

AA: Adipose tissue
BH: Benjamini-Hochberg
FDR: False discovery rate
GWAS: Genome-wide association studies
LD: Latissimus dorsi
MAF: Minor allele frequency
NGS: Next-generation sequencing
PAV: Presence and absence variation
PCV: Principal component analysis
QTP: Qinghai-Tibetan Plateau
SNP: Single nucleotide polymorphism
SV: Structural variation
TAU: Tissue specificity index
TGS: Third-generation sequencing
