# Supplementary figures and images for: Pangenome and multi-tissue gene atlas provide new insights into the domestication and highland adaptation of yaks
Source: J Anim Sci Biotechnol. 2024 May 6;15:64. doi: 10.1186/s40104-024-01027-2 (PMC11071219; doi:10.1186/s40104-024-01027-2)

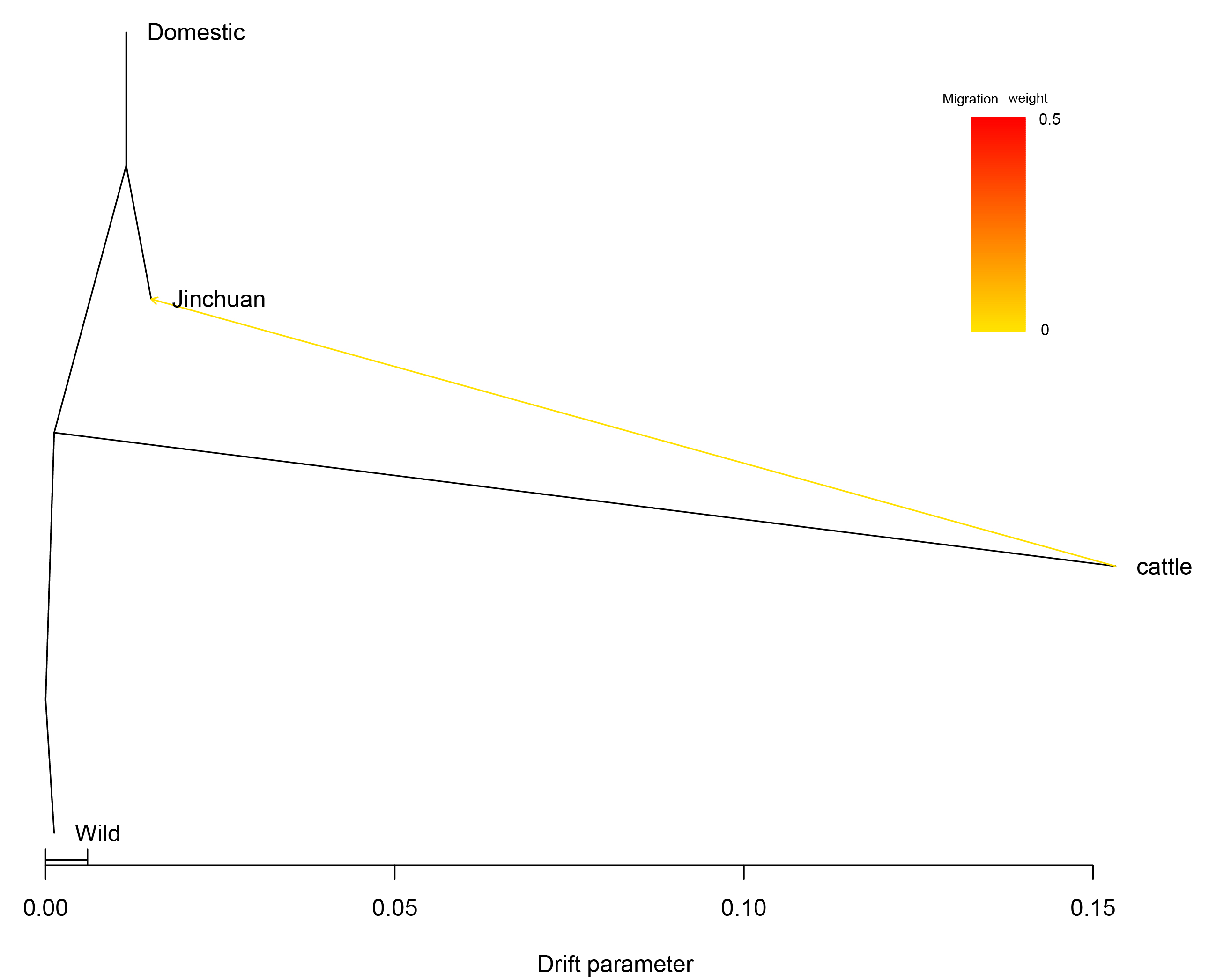

Supplement: Supplementary file 11 — Additional file 11: Fig. S1. The gene flow between yaks and cattle. [file 40104_2024_1027_MOESM11_ESM.jpg]

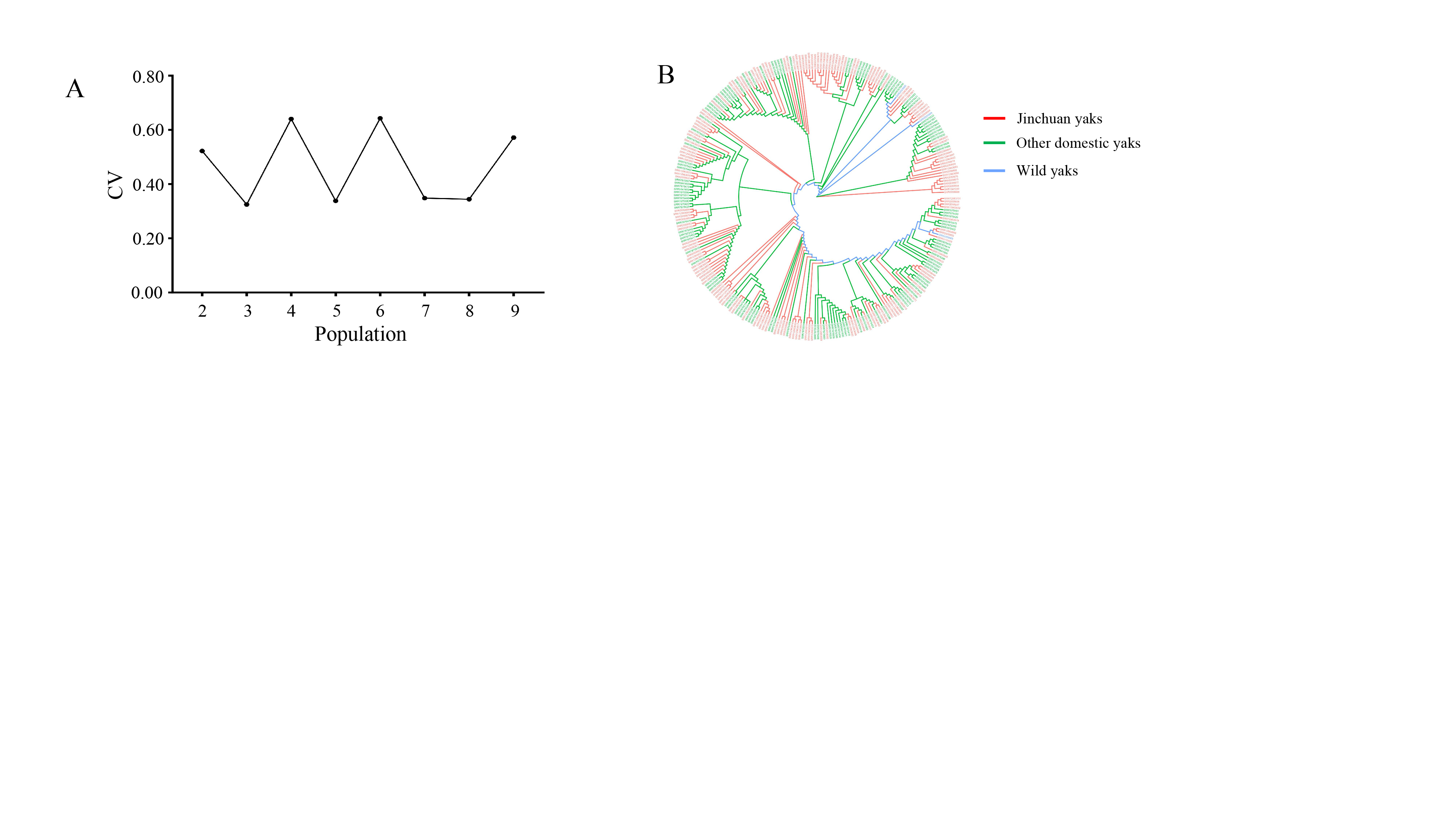

Supplement: Supplementary file 12 — Additional file 12: Fig. S2. A Cross-validation error curves to divide up training data into k-folds; B Phylogenetic tree of yaks constructed based on their mitochondrial genomes. [file 40104_2024_1027_MOESM12_ESM.jpg]

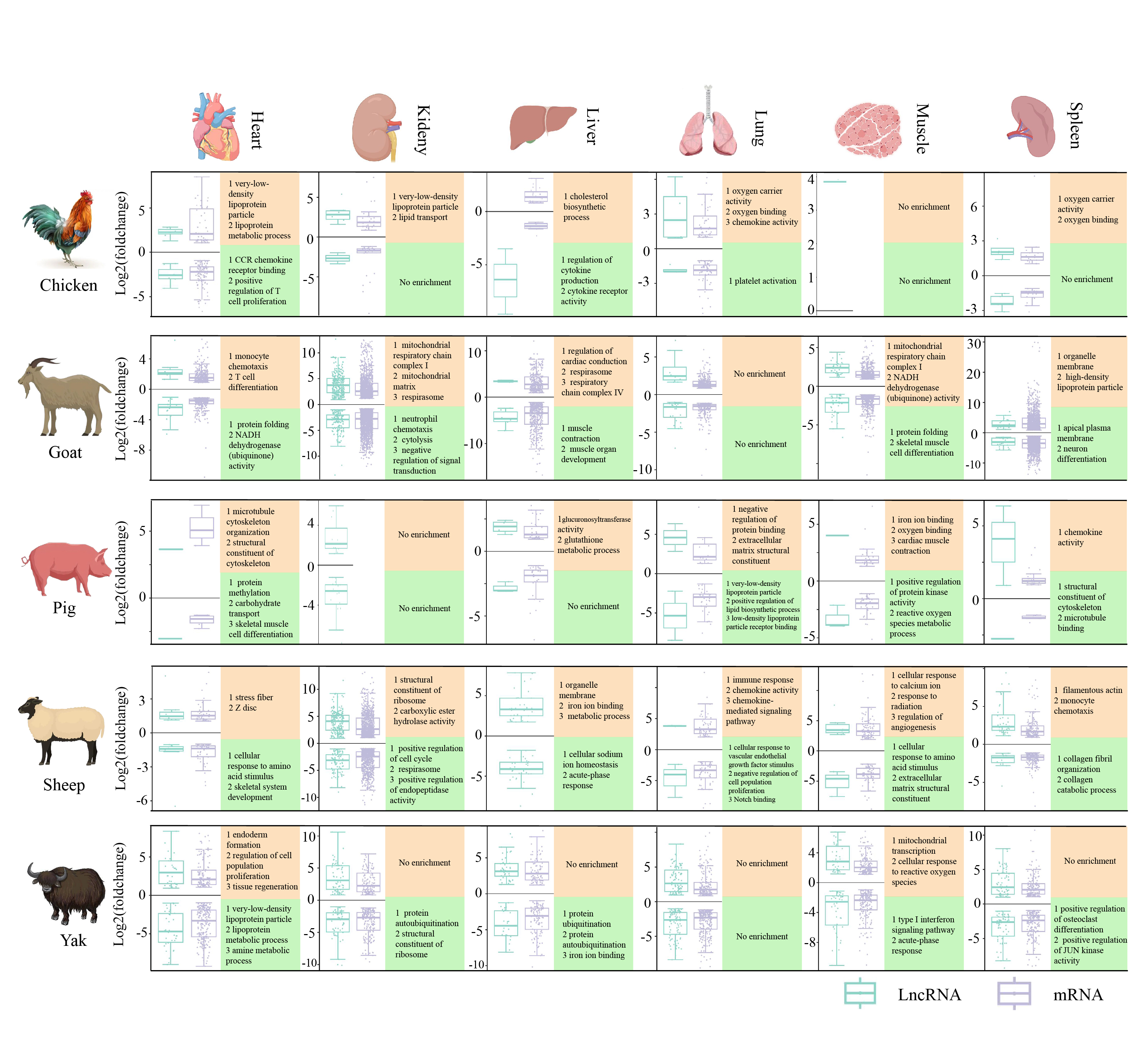

Supplement: Supplementary file 13 — Additional file 13: Fig. S3. The log2 (fold change) values of differentially expressed lncRNA and mRNAs between high and low altitude animals. [file 40104_2024_1027_MOESM13_ESM.jpg]

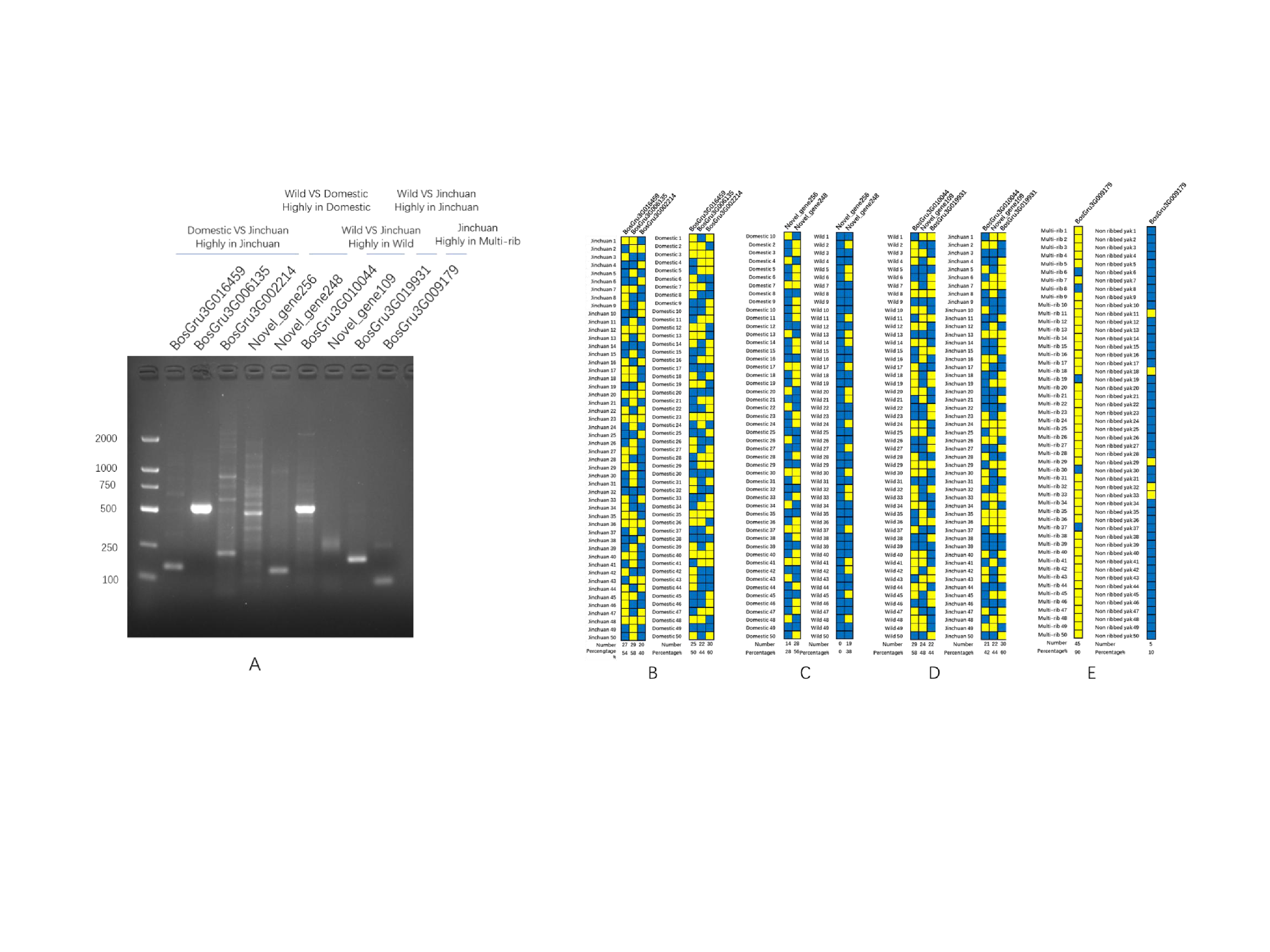

Supplement: Supplementary file 14 — Additional file 14: Fig. S4. A Electrophoresis of the PCR products of BosGru3G016459, BosGru3G006135, BosGru3G002214, Novel_gene256, Novel_gene248, BosGru3G010044, Novel_gene109, BosGru3G019931, and BosGru3G009179. B–E represent heatmaps for BosGru3G016459, BosGru3G006135, BosGru3G002214 in Jinchuan and wild yaks, Novel_gene256 and Novel_gene248 in domestic and wild yaks, BosGru3G019931, BosGru3G010044, Novel_gene109 in Jinchuan and wild yaks, BosGru3G009179 in yaks with 15 pairs of ribs and 14 pairs of ribs, respectively. Yellow indicates the presence of PCR products of corresponding length for the gene in the individual, while blue indicates the absence of PCR products of corresponding length for the gene in the individual. [file 40104_2024_1027_MOESM14_ESM.jpg]
